# Effects of a direct fed microbial (DFM) on broiler chickens exposed to acute and chronic cyclic heat stress in two consecutive experiments

**DOI:** 10.1016/j.psj.2022.101705

**Published:** 2022-01-10

**Authors:** A.H. Sarsour, D.A. Koltes, E.J. Kim, M.E. Persia

**Affiliations:** ⁎ Virginia Tech, Blacksburg, VA, USA; † Iowa State University, Ames, IA, USA; ‡ IFF/Danisco Animal Nutrition, Wilmington, DE, USA

**Keywords:** heat stress, broiler, DFM, intestinal permeability, reused litter

## Abstract

Two consecutive 35 d experiments were conducted to investigate the effects of a multistrain DFM fed continuously to broiler chickens exposed to HS from 28 to 35 d on broiler performance, body composition, ileal digestibility, and intestinal permeability using serum Fluorescein Isothiocyanate Dextran (**FITC-d**) concentration. The treatments were arranged as a 2 × 2 factorial with temperature: Elevated (HS: 33 ± 2°C for 6 h and 27.7°C for the remaining 18 h from 28 to 35 days of age) and Thermoneutral (TN: 22 to 24°C over the entire 24-h day from 28 to 35 days of age) and diet: corn-soybean meal based with and without DFM (3-strain *Bacillus; Enviva PRO*) fed over the entire 35-d period as the two factors. Experimental diets were formulated to meet all nutrient recommendations based on breed standards using a starter (0–10 d), grower (10–21 d), and finisher (21–35 d) period. For each of the 2 experiments, 648 Ross 708 broiler chicks were allotted among the treatments with 9 replicate pens of 18 broilers. Data were analyzed as a 2 × 2 factorial within each experiment in JMP 14. In both experiments, cloacal temperatures were increased (*P* ≤ 0.05) in the broilers subjected to the HS treatment at both 28 d (acute) and 35 d (chronic). Supplementing birds with DFM reduced cloacal temperatures in the Experiment 1 at 28 d, but not at the other time periods. The HS treatment reduced body weight gain and lean tissue accretion from 0 to 35 d in both experiments (*P* ≤ 0.05). In Experiment 2, when the litter was reused BWG was increased by 36 g/bird with supplementation of DFM (*P* ≤ 0.05). Ileal digestibility at 28 d (2 h post HS) was improved with DFM supplementation in both experiments (*P* ≤ 0.05). Serum FITC-d increased with HS at both 28 and 35 d. Serum FITC-d was generally decreased with DFM at 28 d but the response was inconsistent at 35 d. Overall, the results suggest that HS reduced broiler performance and DFM treatment improved intestinal permeability and nutrient digestibility responses to HS in both experiments but did not improve performance until built up litter was used in Experiment 2.

## INTRODUCTION

Although published nearly 20 yr ago, the most recent estimates for economic losses from heat stress in the US poultry industry suggest an annual 128-million-dollar loss (St. Pierre et al., 2003). Growing chickens under heat stress (**HS**) conditions will reduce feed intake representing lost potential and further shift those nutrient resources from growth to thermoregulation (Dale and Fuller, 1979). Therefore, understanding and finding mitigation strategies for the negative effects of heat stress has received extensive attention to reduce losses both from an economic as well as an animal welfare perspective.

Elevated temperatures can disrupt thermoregulation and homeostasis of poultry potentially resulting in lost performance and livability. As a response to elevated temperatures, chickens demonstrate behavioral responses such as increasing the surface area of exposed skin by spreading and fluffing their wings, digging into the litter to find a cooler surface, decreased activity and feed intake, and increased water intake (Mashaly et al., 2004; Quinteiro-Filho et al., 2010; Mack et al., 2013). As birds lack sweat glands, they use the panting mechanism with increased respiration rate which will result in increased water evaporation from the surface of the lungs reducing body temperature (Teeter et al., 1985). The increased respiration rate will result in excessive CO_2_ losses which results in a reduction of bicarbonate (**HCO_3_**), a potent buffer and the primary mode of CO_2_ transportation in the blood, resulting in increased blood pH, or respiratory alkalosis (Koelkebeck and Odom, 1994; Barrett et al., 2019). The increased energy demand from active cooling methods (panting) during heat stress can also cause an increase in reactive oxygen species which can cause DNA damage, lipid peroxidation, and protein oxidation (Quinteiro-Filho et al., 2010). Heat stress can disrupt tight junction proteins in the intestine increasing intestinal permeability of pathogens and metabolites into circulation causing further loss of performance or even disease (Quinteiro-Filho et al., 2010; Wu et al., 2018). Heat stress has, also, been associated with changes in the microflora of the intestine including a reduction in *Lactobacillus* and *Bifidobacterium* and an increase in coliforms and *Clostridium* (Song et al., 2014). These responses to elevated environmental temperature can result in decreased performance, poor feed efficiency, and increased mortality in broilers (Deeb et al., 2002; Imik et al., 2012; Sohail et al., 2012).

One of the possible strategies to ameliorate the negative effect of HS is supplementing direct fed microbials (**DFM**) to poultry diets. These live and beneficial microbes have become a more common feed supplement in the poultry industry as an alternative to growth promoting antibiotics and in antibiotic free production systems. The supplementation of DFMs can modulate the microflora within the host resulting in an improved intestinal balance and health of the bird (Fuller, 1989). Continuously feeding DFMs has resulted in an altered litter microbial community (Pedroso et al., 2013; Li et al., 2014). *Bacillus* spp., a commonly fed DFM, can increase the production of digestive enzymes which can enhance nutrient digestibility as well as inhibit pathogenic bacteria such *Clostridium perfringes* and *E. coli* (Wang et al., 2015; Whelan et al., 2019). Additionally, DFMs can improve intestinal morphological measurements such as villus height and villus height to crypt depth ratio indicating an increased available surface area for absorption of nutrients (Forte et al., 2018; Song et al., 2014). In laying hens, *Bacillus licheniformis* has been reported to reduce serum corticosterone which indicates a reduced stress response after 6 d of HS with the supplementation of a Bacillus-based DFM in the feed ( Deng et al., 2012). This reduction in corticosterone was associated with a reduction in interleukin-1 and tumor necrosis factor α in the blood with supplementation of the DFM during HS. These cytokines have been linked to a reduction in feed intake and increased resting energy expenditure, gluconeogenesis, and glucose oxidation (Klasing, 1988). Consequently, the inclusion of a DFM during heat stress might alleviate the stress response during HS by reduction in cytokine production and exclusion of intestinal pathogens improving live performance and livability.

The working hypothesis was that supplementing a DFM to broiler chickens exposed to a cyclic heat stress would increase growth performance and energy digestibility while decreasing intestinal permeability resulting in altercations of litter. Therefore, the objectives of the current experiments were to determine the effects of a DFM on broilers raised over 2 consecutive flocks and exposed to a cyclic HS on broiler performance, cloacal temperature, body composition, ileal energy digestibility, litter bacterial counts, and intestinal permeability as indicated by serum FITC-d

## MATERIALS AND METHODS

### Broiler Management

**Figure 1:**
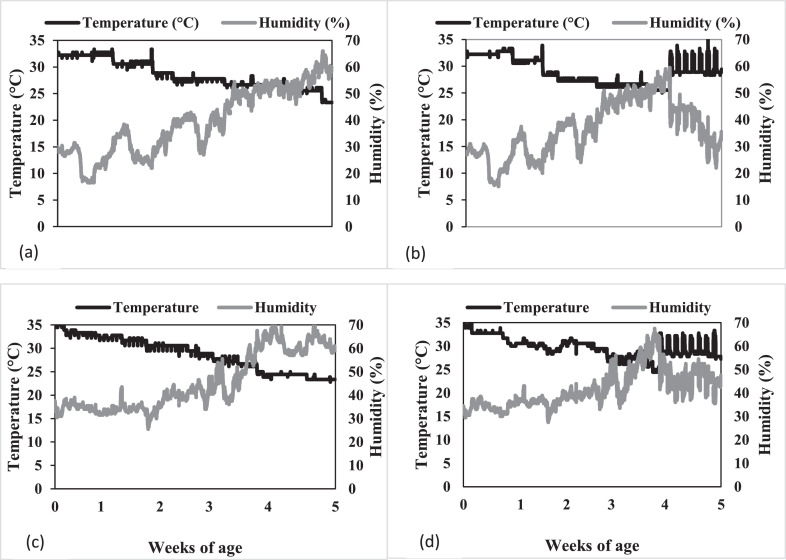
Temperature and relative humidity data from two different rooms in two consecutive experiments logged every 5 min in which broilers were raised to 35 d of age and exposed to HS from 28 to 35 d of age. Experiment 1: (a) Thermoneutral (b) Heat stress. Experiment 2: (c) Thermoneutral (d) Heat stress.

All animal procedures were approved by the Institutional Animal Care and Use Committee at Virginia Tech (Blacksburg, VA). Two consecutive 35 d experiments were conducted to investigate the effects of a multistrain DFM (3-strain *Bacillus; Enviva PRO*) on broiler chickens exposed to HS from 28 to 35 d. The treatments were arranged as a 2 × 2 factorial with temperature: HS (33 ± 2°C for 6 h and 27.7°C for the remaining 18 h from 28 to 35 d of age) and TN (between 22 and 24°C for 28 to 35 d of age) and supplementation with DFM over the entire 35-d growing period as the 2 experimental factors. For both experiments, the temperature treatment were allotted to a room and 648 Ross 708 broiler chicks were allotted to the 2 diet treatment within each room with 9 replicate pens of 18 broilers. Stocking density was maintained at 548 cm^2^/bird from 0 to 28 d of age and 823 cm^2^/bird from 28 to 35 d of age in the 0.923 × 1.07-meter pens. Broilers were provided ad libitum access to experimental feed and water. Until HS treatments were initiated, temperatures were maintained according to breeder specifications based on the age of the birds which ranged from 30°C at placement to 22°C at 28 d of age (Aviagen, 2018). From 28 to 35 d of age, the above noted temperature treatments were applied using constant flow of room temperature air and a heater (TPI Corporation model# F3F551QT, Gray, TN). Ventilation was maintained using mixer fans to minimize difference in temperature across the length of the room. The room temperature was able to reach the target temperature within 20 min of heat exposure. Temperature and humidity were monitored and recorded in each room throughout the experiment (Figure 1). Continuous lighting was provided from 0 to 3 d of age, then the lighting was adjusted to provide 20 h of light and 4 h of darkness (02:00 till 06:00) from 3 to 35 d of age according to the commercial management guide. Heat stress was initiated 2 h after the lights turned on from 28 to 35 d of age. Health checks occurred twice daily and when mortality was discovered, they were removed from the pen, weighed, and recorded. Clean pine shavings were used in Experiment 1, but the same litter was reused in Experiment 2. The litter was mixed and allowed to dry within the same pen to maintain consistency due to possible differences in the litter due to DFM treatment. There was a 7-d downtime period between experiments.

### Diet Formulation

A single experimental starter (0–11 d), grower (11–21 d), and finisher (21–35 d) diet was formulated to meet or exceed the nutrient recommendation of breeder recommendations (Table 1: Aviagen, 2018). A basal diet was generated and then equally split and 500 g/ton of a multistrain DFM (3-strain *Bacillus; Enviva PRO*) was added to the treatment diet and the other left alone to generate the control diet for each dietary phase. Finisher diets were formulated with 0.30% titanium dioxide as an indigestible marker. Starter diets were provided in crumble form and grower and finisher diets in pelleted form. Feed samples were collected and sent to the IFF/Danisco Animal Nutrition lab (Wilmington, DE) for detection of *bacillus* bacteria.

**Table 1:** Formulation and nutrient profile of experimental diets for starter (0–11 d), grower (11–21 d), and finisher (21–35 d) diets fed to Ross 708 broilers and exposed to elevated environmental temperatures from 28 to 35 d^1^.

Ingredient	Starter	Grower	Finisher
	———————–(%)———————–		
Corn	57.10	60.18	61.68
Soybean meal (48% CP)	33.00	27.00	21.00
Dried distillers grains w/ solubles	4.00	5.00	6.00
Poultry byproduct meal	2.00	4.00	6.00
Soy oil	0.10	0.49	1.84
Sodium chloride	0.15	0.20	0.15
Sodium bicarbonate	0.30	0.14	0.18
DL-Methionine	0.33	0.27	0.24
L-Lysine•HCL	0.19	0.14	0.16
L-Threonine	0.13	0.20	0.06
Limestone	1.04	1.01	0.98
Dicalcium phosphate	0.91	0.75	0.59
Phytase^2^	0.02	0.02	0.02
Choline chloride (60%)	0.10	0.10	0.10
Titanium dioxide^3^	—	—	0.30
Vitamin premix^4^	0.63	0.50	0.50
Nutrient profile^5^			
Crude protein	22.50 (21.81)	21.50 (21.25)	20.44 (21.00)
ME, kcal/kg	2,950	3,060	3,160
Calcium	0.90	0.90	0.90
Nonphytate phosphorus	0.45	0.45	0.45
Crude fat	3.60 (3.40)	4.93 (4.16)	6.34 (4.68)
Fiber	2.80 (2.70)	2.76 (2.40)	2.53 (2.10)
Dig. Met	0.62	0.56	0.53
Dig. Cys	0.33	0.31	0.30
Dig. Met+Cys	0.95	0.87	0.83
Dig. Lys	1.28	1.15	1.06
Dig. His	0.52	0.50	0.47
Dig. Trp	0.22	0.20	0.19
Dig. Thr	0.86	0.77	0.71
Dig. Arg	1.29	1.23	1.14
Dig. Iso	0.83	0.79	0.73

1 DFM was added on top of basal diet at 500 g/ton (1.50E+5 CFU/g). DFM supplemented diet analysis ranged between 1.41 and 1.60E+5, while control diets ranged from 6.35E+3 to 2.75E+4 for starter, grower and finisher diets.

2 Axtra Phy (500 FTU/kg) was formulated to provide 0.10% of nPP and calcium.

3 Titanium dioxide was added as an inert marker for digestibility determination.

4 Provided per kg of diet: vitamin A, 1,320,000 IU; vitamin D3, 440,000 ICU; vitamin E, 2,860 IU; menadione, 176 mg; biotin, 6.6 mg; vitamin B12, 1.9 mg; choline, 71.5 g; niacin, 6.6 mg; pantothenic acid, 1.8 g; selenium, 40 mg; riboflavin, 880 mg; Cu, 4.4 g; Fe, 45 g; I, 135 mg; Mn, 44 g; Zn, 44 g; Co, 4.4 g.

5 Values within parenthesis are analyzed values for complete diets.

### Cloacal Temperature and Blood Chemistry

Cloacal temperature was measured at 28 and 35 d of age in each experiment from 5 broilers per pen 2 to 3 h after the initiation of HS. Cloacal temperature measurement was accomplished by inserting a thermometer (DeltaTrak MDL11064, Pleasanton, CA) 2 cm into the cloaca of each broiler until temperature reading was stable for 10 s. One mL of blood was collected from the brachial vein from 1 broiler per pen approximately 2 to 3 h after the initiation of HS. Blood collection was accomplished using a 3 mL syringe with a 23-gauge × 1.6 cm needle with EDTA as an anticoagulant. The collected blood was immediately analyzed using an Abaxis iSTAT handheld analyzer with a CG8+ cartridge (Abaxis item number 600-9001, Union city, CA). Analyzed parameters included ionized calcium (**iCa**), pH, partial pressure of carbon dioxide (**PCO_2_**), partial pressure of oxygen (**PO_2_**), total carbon dioxide (**TCO_2_**), and bicarbonate (HCO_3_).

### Broiler Performance and Body Composition

Individual body weights and pen feed offered and refused were measured at hatch, 11, 21, 28, and 35 d of age including all feed changes and the initiation of heat stress. Body weight gain and feed intake were calculated by difference between final weight and initial weight. Body weight gain and feed intake by pen along with mortality weight was used to calculate mortality corrected feed conversion ratio (**FCRm**) expressed as a ratio of feed consumed to weight gained by adding the pen mortality body weight gain to pen bird body weight gain. Body weights, feed intake, and mortality corrected feed conversion ratio were calculated and analyzed over the 0 to 28, 28 to 35 and 0 to 35 d periods. After placement of chicks on experimental treatments, 30 remaining chicks were euthanized and slowly frozen in −20°C for body composition analysis. The morning after the experiment ended on d 35, five broilers per pen were euthanized, defeathered, and frozen for later analysis. Both the d old and 36-day-old sampled carcasses were allowed to come to room temperature for Dual-energy X-ray Absorptiometry (**DXA**) with a Lunar Prodigy machine (GE Lunar, GE Healthcare, Waukesha, WI). These scans were utilized to measure fat and lean content of carcasses. The percentage of carcass fat and lean was used with body weight to calculate body fat and lean tissue at hatch and 36 d of age. The differences between the 2 sampling days were divided by the number of days to calculate a daily fat and lean accretion value between hatch and 36 d (Tran et al., 2021).

### Ileal Energy Digestibility

Five broilers from each replicate pen were randomly selected and euthanized on d 28 and 35 for ileal digesta collection 2 to 3 h after the initiation of HS and 4 to 5 h after the lights were turned on. Contents from the posterior half of the ileum as defined by Meckel's diverticulum to the ileal-cecal junction were collected by flushing the contents using distilled water. The ileal contents were pooled by pen and collected into WHIRL-PAK bags and frozen until analysis. Ileal samples were dried in a forced air oven at 65°C for 24 to 48 h. Pooled feed samples taken after feed pelleting were used for digestibility determination. Dried ileal content and feed were ground using a cyclone mill with a 2-mm screen. Gross energy was measured in duplicate using a Parr 6400 bomb calorimeter. Feed and ileal samples were digested in sulfuric acid to determine amount of titanium in the sample using the methods of Short et al. (1996). Samples were analyzed by inductively coupled plasma atomic emission spectrometer at 336 nm to determine the concentration of titanium. Ileal energy digestibility was calculated using the following equation (Siegert et al., 2021).

$$Ileal\;energy\;digestibility\;\left(\%\right)=100-100\;\times \;\left(\frac{TiO2\;in\;feed}{TiO2\;in\;digesta}\times \frac{Gross\;energy\;of\;digesta}{Gross\;energy\;of\;feed}\right)$$

### Intestinal Permeability

Serum concentrations FITC-d was used to estimate intestinal permeability 2 h after HS exposure on d 28 and 35 using the methods of Baxter et al. (2017). Briefly, one broiler was randomly selected per replicate pen and was orally gavaged with FITC-d (8.32 mg/kg body weight) dissolved in double distilled water 1 h before the same chick was sampled for blood collection from the brachial vein. These blood samples were stored in serum tubes and allowed to clot for 4 h at room temperature in dark conditions. Serum was isolated from the blood by centrifuging tubes at 2,000 × *g* for 10 min at 4°C. Serum was then removed and diluted 1:5 in sterile 0.9% saline to a total volume of 100 μL in 96-well flat bottom black plate. FITC-d was measured at an excitation wavelength of 485 nm and an emission wavelength of 528 nm using multimode plate reader (Infinite M200 Pro, Tecan, Morrisville, NC). Serum fluorescent concentrations were then determined using a standard curve and sera of chickens not given FITC-d before blood collection, but directly spiked in the sera.

### Statistical Analysis

Data were analyzed as a 2 × 2 factorial within each experiment in JMP 14. Student's *t* test was used to separate the significant least squares main effect and interaction means with the probability set at *P* ≤ 0.05. Diets were randomly allotted to blocks within rows of pens that were used as the random variable in the analyses.

## RESULTS AND DISCUSSION

### Cloacal Temperature and Blood Chemistry

In order to confirm the HS, cloacal temperatures were measured at the acute and chronic stages of heat stress 2 to 3 h after heat stress was initiated (Table 2). No interactions were observed on cloacal temperature in either experiment or time point during HS (*P* > 0.05). HS resulted in elevated cloacal temperatures both at 28 and 35 d of age (*P* ≤ 0.01) confirming the exposure of birds to HS. In the first experiment, there was a reduced cloacal temperature at 28 d during the acute response by the supplementation of the DFM (*P* ≤ 0.05). No differences were noted at 35 d in the first experiment or at either 28 or 35 d in the second experiment with the DFM treatment (*P* > 0.05). Previously, Ross 708 broiler subjected to a cyclic HS at 32°C for 10 h per day demonstrated a reduction in panting and wing spreading with the supplementation of a *Bacillus subtillis* based DFM (Wang et al., 2018). The inconsistent response in these experiments suggest that DFM treatment can possibly alter HS responses, but several factors might be able to alter the response including strain of microbes utilized, environmental conditions including intensity and length of heat exposure, relative humidity, size, and age of the broilers.

**Table 2:** The effect of direct fed microbial (DFM) supplementation on cloacal temperatures (measured 2 h after daily heat exposure on d 28 and 35) of age of broilers exposed to acute HS at 28 d and chronic heat stress at 35 d of age in 2 consecutive experiments using built up litter^1^.

Temperature^2^	Diet^3^	Experiment 1: Clean pine shavings		Experiment 2: Reused litter	
		28 d	35 d	28 d	35 d
		^_____________________________________^°C ^___________________________________^			
TN		41.7^b^	41.8^b^	41.6^b^	41.6^b^
HS		43.5^a^	42.7^a^	42.9^a^	43.2^a^
Pooled SEM		0.05	0.04	0.05	0.07
	Control	42.7^a^	42.3	42.2	42.5
	Control + DFM	42.4^b^	42.1	42.3	42.3
Pooled SEM		0.05	0.04	0.05	0.07
*P*-value^4^					
Temperature		**≤ 0.01**	**≤ 0.01**	**≤ 0.01**	**≤ 0.01**
Diet		**≤ 0.01**	0.27	0.45	0.08
Temperature × Diet		0.28	0.23	0.81	0.74

1 Values are means from 1 bird per pen from 9 replicate pens from each interaction or 18 per main effect.

2 Thermoneutral = continuous 22 to 24°C; Elevated received 33°C for 6 h and 27.7°C for the remaining 18 h daily.

3 DFM, direct fed microbial. ^4^Boldface indicates significant *P*-value. ^a-b^Values in a column without common supperscript letter are different (*P* ≤ 0.05).

**Figure 2:**
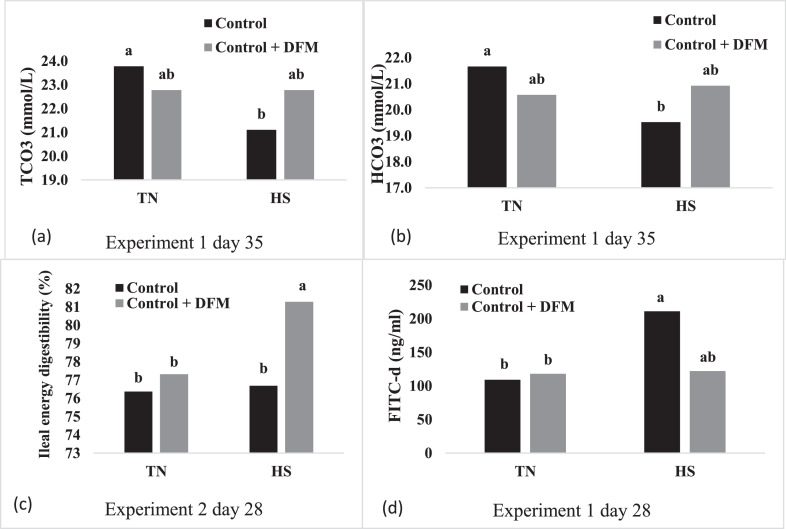
Graphs of significant interactions between temperature and diet. The effect of DFM supplementation on (a) Total carbon dioxide (TCO_2_) in the blood in the Experiment 1 at 35 d of age (*P* = 0.03; SEM = 0.47), (b) Bicarbonate (HCO_3_) in the blood in Experiment 1 at 35 days of age (*P* = 0.05; SEM = 0.49), (c) Ileal energy digestibility in Experiment 2 at 28 d of age (*P* ≤ 0.01; SEM = 0.57), and (d) Fluorescein isothiocyanate dextran (FITC-d) at 28 d of age in Experiment 1 of broiler chickens exposed to heat stress from 28 to 35 d (*P* = 0.04; SEM = 0.22).

No significant interactions were observed for blood chemistry at 28 d (acute bird response to HS) in Experiment 1 (Table 3). Heat stress resulted in a decrease in PCO_2_ (*P* ≤ 0.01) and an increase of blood pH (*P* ≤ 0.01). Furthermore, there was an observed decrease in ionized calcium (iCa) with HS compared to TN treated broilers (*P* ≤ 0.01). Previous research has indicated that elevated blood pH results in the binding of iCa to proteins such as calbindin reducing availability of Ca to the bird (Etches et al., 2008). Broilers were generally not able to adapt to the HS after 7 d of exposure as at 35 d as PCO_2_ was still reduced (*P* ≤ 0.01), and blood pH trended to be elevated (*P* = 0.06). Furthermore, there were two interactions observed for HCO_3_ and total blood carbon dioxide (TCO_2_) at 35 d of age. Bicarbonate and TCO_2_ were both reduced with HS compared to TN without DFM supplementation (Figure 2; *P* ≤ 0.05). A prebiotic and probiotic mixture supplemented to Ross 708 broilers subjected to a cyclic HS at 32°C for 10 h per day from 15 to 42 d of age reduced behaviors associated with heat loss including panting and wing spreading (Mohammed et al., 2018). Heat stress can alter the neuroendocrine system activity which can result in the activation of the HPA axis resulting in higher corticosterone in the blood resulting in increased stress responses and loss of performance (Quinteiro-Filho et al., 2012). It was speculated that the reduction in behaviors such as panting and wing spreading was mediated through the microbiome resulting in improved regulation of the hypothalamic-pituitary-adrenal axis and reduced stress responses (Mayer et al., 2015). An early experiment conducted with Arbor Acre chicks subjected to a continuous HS at 32°C for 3 wk reported a decrease in PCO_2_ and HCO_3_ which resulted in a higher pH in the blood (Teeter et al., 1985). An experiment conducted previously with a leghorn line exposed to a continuous HS at 38°C for 4 h and then maintained at 35°C from 14 to 41 d of age reported a reduction in PCO_2_ along with a decrease in pH but no difference was observed on HCO_3_ or iCa (Wang et al., 2018). Another study where broilers were subjected to an acute HS at 35°C for 8 h at 31 d of age reported a reduction PCO_2_ and HCO_3_ which resulted in an increase in the pH of the blood but no reported effect on iCa (Beckford et al., 2020). These results were consistent with our findings for PCO_2_ and pH but the HCO_3_ responses were more complex as they differed between the acute and (28 d response) and the chronic (35 d response). Previous reports have suggested that this may be due to birds efficiently reabsorbing bicarbonate from the blood (Toyomizu et al., 2005).

**Table 3:** Effects of DFM supplementation on the blood chemistry (measured 2 h after daily heat exposure on d 28 and 35) of broiler chickens raised on clean pine shavings and exposed to heat stress from 28 to 35 d, Experiment 1^1^.

Temperature^2^	Diet^3^	pH		PCO_2_		HCO_3_		TCO_2_		iCa^4^	
		28 d	35 d	28 d	35 d	28 d	35 d	28 d	35 d	28 d	35 d
				^_____^ mm Hg ^_____^		^___________________^ mmol/L ^_________________^					
TN		7.28^b^	7.10	50.5^a^	67.9^a^	23.9	21.1	25.4	23.2	1.39^a^	-
HS		7.44^a^	7.14	35.2^b^	58.8^b^	23.5	20.2	24.5	21.9	1.29^b^	-
Pooled SEM		0.018	0.016	2.00	2.00	0.61	0.43	0.59	0.44	0.016	-
	Control	7.36	7.11	43.2	65.2	24.1	20.6	25.4	22.4	1.35	-
	C + DFM	7.35	7.14	42.5	61.6	23.4	20.7	24.6	22.7	1.34	-
Pooled SEM		0.018	0.016	2.00	2.00	0.61	0.43	0.59	0.44	0.016	-
*P*-value^5^											
Temperature		**≤ 0.01**	0.06	**≤ 0.01**	**≤ 0.01**	0.64	0.15	0.30	0.05	**≤ 0.01**	**-**
Diet		0.78	0.33	0.33	0.14	0.44	0.80	0.38	0.66	0.63	-
Temperature × Diet		0.24	0.60	0.60	0.51	0.17	**0.05**	0.13	**0.03**	0.63	-

1 Values are means from 1 bird per pen from 9 replicate pens from each interaction or 18 per main effect.

2 Thermoneutral = continuous 22 to 24°C; HS (heat stress) received 33°C for 6 h and 27.7°C for the remaining 18 h daily.

3 DFM, direct fed microbial.

4 Value for iCa were not within the detectable range at 35 days of age. ^5^Boldface indicates significant *P*-value. ^a-b^Values in a column without a common superscript letter are different (*P* ≤ 0.05).

In Experiment 2, when broilers were raised on previously used litter, no interactions were observed for blood chemistry at either 28 or 35 d of age (Table 4; *P* > 0.05). Mostly consistent with Experiment 1, the acute response to HS reduced blood PCO_2_, TCO_2_, and HCO_3_ (*P* ≤ 0.01) resulting in an increase in blood pH (*P* ≤ 0.05). At 35 d of age after a 7-d chronic exposure to HS continued to result in a decreased blood PCO_2_, TCO_2_, and HCO_3_ (*P* ≤ 0.01), but pH was better regulated (*P* > 0.05). No significant effects of the DFM were observed on blood gas chemistry either in the acute phase at 28 d or after chronic HS exposure at 35 d of age (*P* > 0.05). Comparisons between acute and chronic HS effects in broilers have been limited, but in laying hens, birds adapt to the HS and the pH and pO2 do go back to normal levels after 4 wk of HS (Barrett et al., 2019), however, the PCO_2_, HCO_3_, and TCO_2_ were still reduced as the hen were still using panting as a major cooling mechanism. There are several potential explanations for the differences in laying hen and broiler responses to chronic HS including differences in adaptation time, age at exposure, growth rate, feed intake, and body size.

**Table 4:** Effects of DFM supplementation on the blood chemistry (measured 2 h after daily heat exposure on d 28 and 35) of broiler chickens raised on clean pine shavings and exposed to heat stress from 28 to 35 d, Experiment 2^1^.

Temperature^2^	Diet^3^	pH		PCO_2_		HCO_3_		TCO_2_		iCa	
		28 d	35 d	28 d	35 d	28 d	35 d	28 d	35 d	28 d	35 d
				^___^ mm Hg ^___^		^___________________^ mmol/L ^___________________^					
TN		7.42^b^	7.41	35.0^a^	38.5^a^	22.8^a^	23.6^a^	23.8^a^	24.7^a^	0.56	0.54
HS		7.51^a^	7.41	26.8^b^	32.6^b^	20.7^b^	20.4^b^	21.6^b^	21.4^b^	0.41	0.62
Pooled SEM		0.026	0.017	1.78	1.71	0.50	0.39	0.52	0.41	0.071	0.075
	Control	7.45	7.42	32.0	33.6	21.5	21.5	22.5	22.5	0.54	0.57
	C + DFM	7.49	7.39	30.0	37.5	21.9	22.5	22.8	23.6	0.41	0.60
Pooled SEM		0.026	0.017	1.78	1.71	0.50	0.39	0.52	0.41	0.071	0.075
*P*-value^4^											
Temperature		**0.03**	0.85	**≤ 0.01**	**0.02**	**≤ 0.01**	**≤ 0.01**	**≤ 0.01**	**≤ 0.01**	0.40	0.53
Diet		0.27	0.39	0.40	0.12	0.55	0.11	0.65	0.08	0.65	0.79
Temperature × Diet		0.08	0.27	0.09	0.12	0.75	0.25	0.88	0.22	0.46	0.11

1 Values are means from 1 bird per pen from 9 replicate pens from each interaction or 18 per main effect.

2 Thermoneutral = continuous 22 to 24°C; HS (heat stress) received 33°C for 6 h and 27.7°C for the remaining 18 h daily.

3 DFM, direct fed microbial. ^4^Boldface indicates significant *P*-value. ^a-b^Values in a column without a common superscript letter are different (*P* ≤ 0.05).

### Broiler Performance and Body Composition

There were no interactions observed among DFM and HS for BWG or FCRm in either experiment (Table 5; *P* > 0.05). No differences were observed at 28 d of age, before application of any HS treatments) between the birds raised in the TN and HS rooms over both experiments (*P* > 0.05). As expected, HS resulted in a reduction in BWG from 28 to 35 d of age in both experiments (130 and 64 g/bird, respectively; *P* ≤ 0.01). Mortality corrected feed conversion ratio was worsened in broilers that were subjected to elevated temperature from 28 to 35 d of age in Experiment 2 (*P* ≤ 0.01). Previous research has shown some combination of reduction in performance including reduction in BWG and/or worsening of FCR when broilers were subjected to heat stress. One experiment conducted with male broilers that were subjected to cyclic HS from 35 to 41 d of age at either 31 or 36°C for 10 h per day then reduced to the thermoneutral comfort level for the remaining 14 h observed a reduction in BWG with both the HS temperatures, however the FCR was only worsened when the HS temperature was maintained at 36°C compared to the control (Quinteiro-Filho et al., 2010). Additionally, a 31°C HS temperature was found to reduce BWG from 35 to 41 d of age without differences in FCR compared to the control group (Quinteiro-Filho et al., 2012). Another experiment conducted with Ross 708 mixed sex chicks that were heat stressed continuously from 0 to 42 d of age observed a reduction in BWG and an increase in FCR between 0 and 42 d of age but only observed the reduction in BWG from 0 to 21 d of age (Sohail et al., 2012). Taken together the current and previous results suggest that BWG is more sensitive to HS than FCR. This could be due to an early response to HS being a reduction in feed intake to reduce metabolic heat production reducing feed intake, but not altering metabolism (Lagana et al., 2007). Once HS is more severe, metabolic mechanisms begin to break down and FCR is worsened (Zhang et al., 2020).

**Table 5:** Effects of DFM supplementation on body weight gain and mortality corrected feed conversion ratio (FCRm) of broilers over the 0 to 35 d period when exposed to heat stress from 28 to 35 d^1^.

Temperature 2	Diet^3^	^___________^ Experiment 1 with clean pine shavings ^___________^						^_______________^ Experiment 2 with reused litter ^_______________^					
		Body weight gain			FCRm			Body weight gain			FCRm		
		0 to 28	28 to 35	0 to 35	0 to 28	28 to 35	0 to 35	0 to 28	28 to 35	0 to 35	0 to 28	28 to 35	0 to 35
		^_______________^ (g) ^_______________^			^_______^ (g feed/g gain) ^_______^			^_______________^ (g) ^_______________^			^_______^ (g feed/g gain) ^_______^		
TN		1,641	665^a^	2,308^a^	1.443	1.603	1.416	1,291	611^a^	1,901	1.473^b^	1.728^b^	1.547^b^
HS		1,605	535^b^	2,140^b^	1.470	1.630	1.450	1,263	547^b^	1,809	1.548^a^	1.970^a^	1.660^a^
Pooled SEM		17	31	44	0.016	0.060	0.022	13	16	20	0.014	0.038	0.012
	Control	1,620	587	2,207	1.467	1.636	1.446	1,257^b^	586	1,843	1.533^a^	1.86	1.628^a^
	C + DFM	1,626	612	2,238	1.445	1.596	1.436	1,296^a^	572	1,868	1.488^b^	1.83	1.579^b^
Pooled SEM		17	31	44	0.016	0.060	0.02	13	16	20	0.014	0.038	0.012
*P*-value^4^													
Temperature		0.15	**≤ 0.01**	**≤ 0.01**	0.28	0.74	0.28	0.13	**≤ 0.01**	**≤ 0.01**	**≤ 0.01**	**≤ 0.01**	**≤ 0.01**
Diet		0.82	0.56	0.89	0.84	0.67	0.35	**0.04**	0.55	0.38	**0.03**	0.60	**≤ 0.01**
Temperature × Diet		0.60	0.65	0.63	0.52	0.39	0.72	0.93	0.62	0.65	0.06	0.88	0.29

1 Values are means from 9 replicate pens from each interaction or 18 per main effect.

2 TN: thermoneutral = continuous 22 to 24°C; HS: heat stress received 33°C for 6 h and 27.7°C for the remaining 18 h daily.

3 DFM, direct fed microbial. ^4^Boldface indicates significant *P*-value. ^a-b^Values in a column without common superscript letter are different (*P* ≤ 0.05).

In Experiment 1, when all pens started with clean pine shavings there were no differences in body weight or FRCm over the 0 to 28 d period, prior to the increase in temperature (*P* > 0.05). This lack of response to DFM treatment in a clean or new litter environment has been reported (Gunal et al., 2006; Shargh et al., 2012; Olnood et al., 2015). Conversely, in the second experiment FCRm over the 0 to 28 d period was worsened in the pens that were exposed to HS in the first experiment. This might suggest that the HS treatments changed the quality of the litter or the litter microbiome resulting in the reduced efficiency. In Experiment 2 with the reused litter, DFM treated birds resulted in an improved FRCm over the 0 to 28 d. In the used litter environment, the DFM might be more effective with more microbial challenge than clean litter (Gil De Los Santos et al., 2005; De Oliveira et al., 2019; Whelan et al., 2019). It is possible that the DFM is altering the litter or litter microbiome resulting in the improved performance although these measurements were beyond the scope of this report. The improvements in BWG and FCRm over the 0 to 28 d period were not carried into the HS period from 28 to 35 d of age resulting in FCRm being the only improvement over the entire 0 to 35 d period (*P* ≤ 0.01). Male Hubbard broilers raised in battery cages and subjected to a cyclic HS (35°C for 5 h and 21°C for 19 h) from 21 to 35 d of age found an improvement in BWG with the supplementation of a *B. subtilis* DFM during HS (Al Fataftah and Abdelqader, 2014). Another experiment utilizing male Cobb broilers raised in battery cages and subjected to a continuous HS at 35°C from 15 to 35 d of age supplemented with a *Lactobacillus* strain probiotic found improved BWG and FCR (Jahromi et al., 2016). Ross 708 broilers subjected to a cyclic HS at 35°C for 10 h and 21°C for the next 14 h from 15 to 43 d of age reported an improvement in BWG and FCR with the supplementation of a *B. subtilis* DFM to broilers raised in floor pens and exposed to HS (Wang et al., 2018). Hubbard broilers subjected to a cyclic HS at 35°C for 8 h and supplemented with a DFM consisting of several *Lactobacillus* strains did not find any effect on BWG but found an improved FCR with supplementation of DFM to broilers in floor pens subjected to HS (Ashraf et al., 2013). Conversely, another experiment utilizing Ross 708 broiler chickens subjected to a cyclic HS for 8 h daily at 35°C from 22 to 42 d of age fed diets containing a *Lactobacillus* strain found no improvement in BWG or feed efficiency (Sohail et al., 2012). A follow-up experiment utilizing Hubbard broiler chickens subjected to a cyclic HS for 8 h daily at 35°C from 22 to 42 d of age fed diets containing the same *Lactobacillus* strain found no improvement in BWG or feed efficiency (Sohail et al., 2013). A recent experiment utilizing Ross 308 broilers subjected to a 10-h cyclic HS at 33°C reported no effect on BWG or FCR when supplementing diets with *B. subtilis* and *B. lichenformis* (Song et al., 2014). The variation in responses to the DFM can be caused by duration of the HS and different modes of action among various DFM. Kazemi et al. (2019) reported that different strains of DFMs have different modes of actions with some acting to improve live performance while others could improve the intestinal microflora and histomorphology for better nutrient absorption.

There were no interactions observed among DFM and HS for lean or fatty tissue accretion in either experiment (Table 6; *P* > 0.05). A reduction in lean and fatty tissue accretion was observed in Experiment 1 when the broilers were subjected to elevated temperature compared to birds held at TN temperatures. However, only lean tissue accretion was reduced in Experiment 2 with no effect observed on fatty tissue accretion. An older report utilizing Arbor Acres males exposed to cyclic 35°C HS for 4 to 6 h resulted in reduced breast weight at 49 d of age but no difference in abdominal fat (Smith, 1993). Similarly, Arbor Acre males that were subjected to a constant HS of 34°C from 35 to 56 d of age reported a reduction in breast meat yield as well as subcutaneous, abdominal, and intermuscular fat compared to a control group that was maintained at 21°C (Lu et al., 2007). The inconsistent response in the current experiment and in the literature suggest that energy deposition may be more complex than lean tissue accretion with factors such as dietary energy, temperature and length of HS exposure, and age of broilers playing a role in the responses.

**Table 6:** Effects of DFM supplementation on the overall lean and fat tissue accretion and ileal energy digestibility (measured 2 h after daily heat exposure on d 28 and 35 to measure acute and chronic responses, respectively) of broiler chickens exposed to heat stress from 28 to 35 d in both experiments^1^.

Temperature^2^	Diet^3^	Experiment 1 with clean pine shavings				Experiment 2 with reused litter			
		Lean tissue accretion	Fat tissue accretion	Ileal Energy Digestibility		Lean tissue accretion	Fat tissue accretion	Ileal energy digestibility	
		0–35 d	0–35 d	28 d	35 d	0–35 d	0–35 d	28 d	35 d
		^__________^ (g/d) ^__________^		^__________^ (%) ^__________^		^__________^ (g/d) ^__________^		^__________^ (%) ^__________^	
TN		48.5^a^	11.5^a^	77.8^a^	78.5	42.6^a^	8.7	76.8	76.9
HS		45.8^b^	10.2^b^	73.4^b^	77.6	40.4^b^	9.0	79.0	77.8
Pooled SEM		0.49	0.17	0.81	0.83	0.52	0.22	0.51	1.50
	Control	47.0	10.7	74.1^b^	77.5	41.8	8.7	76.5	75.5
	Control + DFM	47.2	11.0	77.1^a^	78.6	41.5	8.9	79.3	79.2
Pooled SEM		0.49	0.17	0.81	0.83	0.52	0.22	0.51	1.50
*P*-value^4^									
Temperature		**≤ 0.01**	**≤ 0.01**	**≤ 0.01**	0.37	**0.02**	0.37	≤ 0.01	0.68
Diet		0.72	0.35	**≤ 0.01**	0.31	0.73	0.48	≤ 0.01	0.10
Temperature × Diet		0.84	0.70	0.69	0.38	0.61	0.84	**≤ 0.01**	0.29

1 Values are means from 5 bird per pen from 9 replicate pens from each interaction or 18 per main effect.

2 TN: thermoneutral = continuous 22 to 24°C; HS: heat stress received 33°C for 6 h and 27.7°C for the remaining 18 h daily.

3 DFM, direct fed microbial. ^4^Boldface indicates significant *P*-value. ^a-b^Values in a column without a common superscript letter are different (*P* ≤ 0.05).

### Ileal Energy Digestibility

Ileal samples were taken 2 to 3 h after the initiation of HS instead of fecal samples to capture the immediate effects of acute and chronic heat stress on energy digestibility in an attempt to better understand both digestibility and intestinal health responses. No interactions were observed in Experiment 1 (Table 6; *P* > 0.05), but in Experiment 2 at the 28-d acute period an interaction was noted (Figure 2; *P* ≤ 0.05). The addition of the DFM to broilers under TN conditions had no effect on ileal energy digestibility, but the addition of the DFM to broiler under HS improved digestibility. In Experiment 1, acute HS at 28 d resulted in main effects for both the HS and DFM as HS decreased and DFM increased ileal energy digestibility independently (*P* ≤ 0.05). As the broilers adjusted to the HS at 35 d, there were no significant effects of HS or DFM (*P* > 0.05), although in Experiment 2, under the reused litter conditions, DFM tended to increase ileal energy digestibility (*P* = 0.10). Heat stress has been shown to reduce the activity of digestive enzymes within the intestine mainly amylase and lipase within 2 h of first HS exposure in broilers at 36 d of age (Hao et al., 2012). The activity of amylase and lipase were restored back to pre-HS levels within 10 h of HS exposure which was correlated with an increase in heat shock protein within the intestine. Heat stress effects on digestibility have been mixed. Male Cobb broilers subjected to a constant 32°C from 28 to 46 d increased AME compared to control birds (Keshavarz and Fuller, 1980). Male broilers subjected to a continuous 32°C HS from 45 to 56 d of age increased AME (Geraert et al., 1992). Conversely, continuous 32°C HS of male Cobb 500 broilers resulted in no difference in AME at 31 d of age (Rosa et al., 2007). A more recent experiment used male Cobb broilers under cyclic and continuous HS found no differences in AMEn from 39 to 42 d of age compared to controls (de Souza et al., 2016). The measurement of AME gives both the energy that is directly digested by the bird, but also includes fermentation by microbes and subsequent passive absorption of energy rich volatile fatty acid metabolic waste products of fermentation. Therefore, the comparison of AME to ileal energy digestibility may not be the best comparison as reduced feed intake could increase retention time and alter fermentation adding additional factors that can confound the ability of the broilers to directly digest and absorb energy or nutrients from the diet. In this case, dry matter or amino acid digestibility might be a better comparison to understand the effects of HS on ileal energy digestibility. There are several reports that show continuous HS reduces both dry matter and amino acid and protein ileal digestibility (Geraert et al., 1992; Zuprizal et al., 1993; de Souza et al., 2016). Considering these comparisons, it does appear that HS compromises the ability of broilers to digest and absorb energy and nutrients directly from the diet.

Similar to the current results, DFMs have be reported to have the ability to improve AME in nutrient deficient diets (Weallands et al., 2017; Nusairat and Wang, 2020). Again, this AME comparison may be less valuable as it adds additional factors such as fermentation and increased retention time to consider. More directly, both dry matter and crude protein digestibility were increased in Cobb 500 broilers subjected to a cyclic HS at 36°C for 7 h per day and supplemented with a *Lactobacillus* and *Saccharomyces* based DFM (Attia and Hassan, 2017).

There are several demonstrated modes of actions that would support the ability of DFMs to increase nutrient absorption as demonstrated in the current report. *Bacillus* based DFMs have been reported as a principal source of microbial enzymes including amylases, proteases, lipases, and phytases, potentially increasing enzyme activity to increase nutrient absorption (Lattorre et al., 2016). Furthermore, DFMs have can also improve intestinal morphological measurements such as villus height and villus height to crypt depth ratio resulting in an increased available surface area for absorption of nutrients (Forte et al., 2018; Song et al., 2019).

### Intestinal Permeability

Fluorescein isothiocyanate-dextran (**FITC-d**) has been used widely in the literature as an indicator of intestinal permeability. During HS, disease challenge, or feed restriction there is disruption to tight junction proteins that are located within the epithelial cells in the intestine which allows translocation of pathogens or metabolites into circulation (Maejima et al., 1984; Koh et al., 1996). An interaction was observed at 28 d in Experiment 1 (Figure 2; *P* ≤ 0.05). Heat stress increased serum FITC-d concentration compared to the TN group without DFM but supplementing the DFM in the HS group was able to reduce the FITC-d similar to the TN group regardless of DFM. At 35 d of age, as the broilers adjusted to HS, there were no effects of HS or DFM on intestinal permeability as indicated by serum FITC-d concentration (Table 7; *P* > 0.05). In the second experiment during acute HS at 28 d, HS increased the intestinal permeability compared to TN group (*P* ≤ 0.05), but the addition of DFM did not reduce intestinal permeability as indicated by serum FITC-d concentration (*P* > 0.05). At 35 d, FITC-d serum concentrations were still elevated compared to TN group, but in this case the addition of the DFM tended to reduce the serum FITC-d concentration (*P* = 0.06). Cobb-500 broilers exposed to continuous HS from 21 to 42 d of age showed increased serum FITC-d concentration on both 35 and 42 d (Ruff et al., 2020). Investigation of the effects of a 2 h heat stress at 29 d of age HS on four different breeds of broilers chickens found that 3 of the breeds had higher FITC-d serum concentrations compared to TN groups (Tabler et al., 2020). Although somewhat inconsistent, DFM was able to reduce serum FITC-d concentrations in 2 of the 4 time periods explored in the current research. This is one of the first reports of DFM effects on intestinal permeability in broilers under HS, but DFM treatment has been shown to affect intestinal permeability in other intestinal challenge models. Treatment of broilers with *Bacillus* species have been shown to reduce intestinal permeability 10 d after a *Salmonella* challenge (Adhikari et al., 2019). Cobb 500 broilers infected with *Salmonella typhimurium, Eimeria maxima*, and *Clostridium perfringens* were treated with a *Bacillus*-based DFM resulting in a reduction in serum FITC-d concentrations at 21 d of age compared to a challenged group without DFM (Hernandez-Patlan et al., 2019). Previous data has alluded to the fact that DFMs can increase the transcript abundance expression of tight junction proteins such as JAM2, claudin, and occludin within the intestine which have been linked with reduced the intestinal permeability (Gadde et al., 2017; Zhang et al., 2017).

**Table 7:** Effects of DFM supplementation on Fluorescein Isothiocyanate Dextran (FITC-d) (measured 2 h after daily heat exposure on d 28 and 35 to measure acute and chronic responses, respectively) of broiler chickens exposed to heat stress from 28 to 35 d in both experiments^1^.

Temperature^2^	Diet^3^	Experiment 1: Clean pine shavings		Experiment 2: Reused litter	
		28	35	28	35
		^___________________________________^ng/mL^____________________________________^			
TN		114^b^	104	125^b^	119^b^
HS		167^a^	118	138^a^	128^a^
Pooled SEM		17	5	4	2
	Control	160	112	135	129^a^
	Control + DFM	120	111	128	118^b^
Pooled SEM		17	5	4	2
*P*-value^4^					
Temperature		0.03	0.10	**0.03**	**≤ 0.01**
Diet		0.09	0.83	0.21	**≤ 0.01**
Temperature × Diet		**0.04**	0.81	0.22	0.06

1 Values are means from 1 bird per pen from 9 replicate pens from each interaction or 18 per main effect.

2 Thermoneutral = continuous 22 to 24°C; HS received 33°C for 6 h and 27.7°C for the remaining 18 h daily.

3 DFM, direct fed microbial. ^4^Boldface indicates significant *P*-value. ^a-b^Values in a column without common letter are different (*P* ≤ 0.05).

The cyclic heat stress used in this experiment where broilers were not restored to thermoneutral temperatures after the HS period had a negative impact on performance resulting in reduced BWG, and protein and fat accretion in Experiment 1 and BWG, FCR, and protein accretion in Experiment 2. Cyclic HS resulted in a typical HS response including increased body temperature and the broiler response to this increased temperature using panting to increase evaporative cooling altering blood chemistry and ultimately resulting in blood alkalosis during the acute phase of HS. However, after 1 wk of cyclic HS, blood pH, and pCO_2_ were returning to normal levels which indicates acclimation to elevated temperatures. The same acclimation response could be seen on the response to FITC-d an indicator of intestinal permeability. The effect of the DFM was only apparent on growth performance improvement response when there was a more challenging environment in Experiment 2 after a flock of broilers were raised on the litter which suggests that DFMs might be more beneficial with a more challenging environment. The DFM did not have any effect on performance during the HS, however, DFM did result in improved blood chemistry, ileal energy digestibility, and reduced intestinal permeability when broilers were exposed to a cyclic HS, which may improve long-term health of the broilers, particularly if a second insult, such as a bacterial challenge from reused litter, occurs during or shortly following HS.
